# PfCap380 as a marker for *Plasmodium falciparum* oocyst development in vivo and in vitro

**DOI:** 10.1186/s12936-018-2277-6

**Published:** 2018-04-02

**Authors:** Leslie S. Itsara, Yaxian Zhou, Julie Do, Samrita Dungel, Matthew E. Fishbaugher, Will W. Betz, Thao Nguyen, Mary Jane Navarro, Erika L. Flannery, Ashley M. Vaughan, Stefan H. I. Kappe, Anil K. Ghosh

**Affiliations:** 1 MalarVx, Inc., 307 Westlake Ave N Suite 200, Seattle, WA 98109 USA; 2 Center for Infectious Disease Research, 307 Westlake Ave N Suite 500, Seattle, WA 98109 USA

**Keywords:** *Plasmodium falciparum*, Ookinete, Oocyst, Capsule, PfCap380, Malaria vaccines, Culturing system

## Abstract

**Background:**

Despite the importance of the *Plasmodium berghei* oocyst capsule protein (PbCap380) in parasite survival, very little is known about the orthologous *Plasmodium falciparum* capsule protein (PfCap380). The goal of this work was to study the growth of *P. falciparum* oocysts using PfCap380 as a developmental marker.

**Methods:**

To study *P. falciparum* oocyst development using both in vivo (mosquito-derived) and in vitro (culture-derived) growth conditions, antibodies (polyclonal antisera) were raised against PfCap380. For studies on in vivo oocysts, mature *P. falciparum* gametocytes were fed to *Anopheles stephensi* mosquitoes. For studies on in vitro parasites, *P. falciparum* gametocytes were induced and matured for subsequent ookinete production. Ookinetes were purified and then tested for binding affinity to basal lamina components and transformation into early oocysts, which were grown on reconstituted basal lamia coated wells with novel oocyst media. To monitor in vivo oocyst development, immunofluorescence assays (IFA) were performed using anti-PfCap380 antisera on *Pf*-infected mosquito midguts. IFA were also performed on culture-derived oocysts to follow in vitro oocyst development.

**Results:**

The anti-PfCap380 antisera allowed detection of early midgut oocysts starting at 2 days after gametocyte infection, while circumsporozoite protein was definitively observed on day 6. For in vitro culture, significant transformation of gametocytes to ookinetes (24%) and of ookinetes to early oocysts (85%) was observed. After screening several basal lamina components, collagen IV provided greatest binding of ookinetes and transformation into early oocysts. Finally, PfCap380 expression was observed on the surface of culture-derived oocysts but not on gametocytes or ookinetes.

**Conclusions:**

This study presents developmental monitoring of *P. falciparum* oocysts produced in vivo and in vitro. The anti-PfCap380 antisera serves as an important reagent for developmental studies of oocysts from the mosquito midgut and also from oocyst culture using in vitro methodology. The present data demonstrate that PfCap380 is a useful marker to follow the development and maturation of in vivo and in vitro produced oocysts as early as 2 days after zygote formation. Further in vitro studies focused on oocyst and sporozoite maturation will support the manufacturing of whole sporozoites for malaria vaccines.

**Electronic supplementary material:**

The online version of this article (10.1186/s12936-018-2277-6) contains supplementary material, which is available to authorized users.

## Background

Malaria caused by *Plasmodium* infection is a devastating disease resulting in an annual 212 million cases and 429,000 malaria deaths worldwide. Most deaths occur in Africa (92%), followed by the South-East Asia (6%) and the Eastern Mediterranean (2%) [1]. Several tools including insecticide spray, insecticide-treated bed nets, and anti-malarial drugs have dramatically reduced malaria. However, malaria persists in many countries due to lack of resources, non-compliance, and resistance to insecticides and drugs. In addition to these tools, several vaccine approaches are in development including single-subunit vaccines and live whole-parasite vaccines based on the sporozoite (SPZ) stage. Of the single-subunit vaccines, RTS, S/AS-01 (RTS, S) is the most developed and primarily targets humoral immune responses against the major SPZ surface antigen, circumsporozoite protein (CSP). While RTS, S has completed Phase 3 clinical trials, it did not meet the target goal of providing > 75% efficacy as outlined by the WHO Malaria Vaccine Technology Roadmap [2]. Unlike single-subunit vaccines, whole-SPZ vaccines target humoral and cellular immune responses against considerably more antigens and can provide complete protection against *Plasmodium* infection in mice and humans [3–5]. Whole-SPZ vaccines include live attenuated parasites or WT parasites administered in combination with chloroquine chemoprophylaxis (CPS or CVac). Live attenuated parasites include radiation attenuated sporozoites (RAS) and genetically attenuated parasites (GAP). Attenuated SPZ infect liver cells but are developmentally blocked to prevent blood stage transition, conferring immunity without causing symptoms [3, 4, 6–8].

While whole-SPZ are promising malaria vaccine candidates, a hurdle remains in obtaining large quantities to vaccinate the 2.2 billion people at risk. Current methods of vaccination with SPZ include delivery by mosquito bite and injection of SPZ that are isolated from the salivary glands of infected mosquitoes. These methods are not scalable and isolation from mosquitoes is extremely labour-intensive and costly, limiting large-scale manufacturing and thus commercialization of malaria vaccines based on whole SPZ developed in mosquitoes. A solution to the manufacturing problem of whole SPZ, is an in vitro SPZ platform or culturing system to obtain large quantities of whole SPZ for malaria vaccines.

*Plasmodium falciparum* develops through five stages in mosquitoes including gamete, zygote, ookinete, oocyst, and SPZ. Prior to the gamete stage, gametocytes mature and circulate within the vertebrate host. Mosquitoes become infected by *P. falciparum* after ingesting mature gametocytes during a blood meal taken from a human carrier. After ingestion, gametocytes within the mosquito midgut emerge and transform into gametes that fuse to form a zygote. The zygote undergoes DNA replication and maturation into a motile ookinete that traverses the mosquito peritrophic membrane and mosquito midgut epithelium, and finally embeds between the midgut epithelium and basal lamina on the midgut periphery. Here, the ookinete develops into a sessile oocyst in which many rounds of genome replication occur, and after 10–14 days, mature oocysts produce thousands of SPZ.

Previous in vitro SPZ culturing attempts on several *Plasmodium* species have been reported. SPZ for the avian malaria parasite *Plasmodium gallinaceum* were produced in vitro but SPZ functionality assessed by hepatocyte invasion was not reported [9]. Other studies on the rodent malaria parasites, *Plasmodium berghei* [10] and *Plasmodium yoelii* [11] produced SPZ in vitro, and demonstrated mouse hepatocyte infection and transition to blood stage parasites. For in vitro culturing of *P. falciparum* oocysts and SPZ, very little is known as only a single limited study by Warburg and Schneider describes development of oocysts and SPZ [12]. However, SPZ functionality was not determined as hepatocyte invasion was not addressed [12]. While this publication demonstrates in vitro production of *P. falciparum* oocysts and SPZ, it is the only publication to do so. Many researchers have failed to replicate mosquito-stage culturing of *P. falciparum* and other *Plasmodium* species due to scientific and technical challenges of establishing the culturing systems and due to the complexity of *Plasmodium* mosquito stage development.

To culture SPZ in vitro, one must understand how *Plasmodium* develops within the mosquito and recapitulate those conditions in culture. As oocysts develop, they secrete several proteins, and these secreted proteins along with mosquito-derived factors form a non-lipid bilayer structure surrounding the oocysts called the capsule. An oocyst-derived capsular protein described for *P. berghei*, PbCap380, was found to be is essential for SPZ development [13]. Transcription of the Cap380 gene presumably occurs from a single exon, and the Cap380 protein has a putative N-terminal signal sequence targeting the protein to the secretory pathway for surface localization [13]. Antibodies against PfCap380 would improve in vitro culturing systems by allowing for oocyst-stage detection, quantification, and determination of transformation rates from ookinetes to early oocysts. Prior to these studies, an antibody against PbCap380 was developed, but due to low homology of the immunogenic region between *P. berghei* and *P. falciparum* that was used to raise the anti-PbCap380 antibody, anti-PbCap380 antibody does not cross react with the *P. falciparum* oocyst capsule (unpublished observations). Here, detailed in vitro methodology are presented for production of early *P. falciparum* oocysts along with a reagent (anti-PfCap380 antisera) to study in vivo and in vitro oocyst development. The aims of this study include detecting early oocysts and following the developmental progression of oocysts using PfCap380 as a marker. The present study should enable the standardization of in vitro culture systems that produce the mosquito equivalent stages, (oocysts and SPZ) for scalable production of whole SPZ based malaria vaccines.

## Methods

### Gametocyte culture and mosquito infections

The luciferase expressing strain *P. falciparum* NF54HT-GFP-luc [14] was maintained in complete media (CM), which is Roswell Park Memorial Institute (RPMI) 1640 Media, containing 25 mM HEPES, 2 mM l-glutamine, and 50 μM hypoxanthine (Mediatech, VA) plus 10% human serum (Valley Biomedical, VA). The strain was sub-cultured in CM with 5% O^+^ human erythrocytes (Valley Biomedical, VA) and gametocytes seeded using previously established methods [15–18]. Mature sexual stage gametocytes were induced by allowing continuous growth of cultures without the addition of fresh red blood cells (RBC), as previously described [19]. Briefly, asexual cultures were inoculated at 2% trophozoite parasitaemia and 5% haematocrit. Media was changed daily and thin blood smears were observed beginning on day 9 to determine maturation of gametocytes. Mature gametocytes (day 12–14 post-induction) were fed to 7-day-old *Anopheles stephensi* mosquitoes to initiate infection. The mature gametocyte culture was diluted 1:1.8 with fresh whole blood and then diluted 1:1 with human serum. Mosquitoes were allowed to feed for 20 min and were then incubated at 27 °C and 75% humidity and given 8% dextrose with 0.05% para-aminobenzoic acid (Sigma-Aldrich, MO). To check midgut infection of *P. falciparum*, mosquitoes were dissected between days 2–9 after gametocyte infection.

### Ookinete culture

Gametocyte culture was seeded and matured as described above. Media was changed daily and tri-gas (90% N_2_, 5% CO_2_, 5% O_2_) added to the flask for ~ 90 s. Emergence of male gametes (exflagellation) was measured between days 12–16 and the culture was considered mature when ≥ 1 exflagellation event per field was observed in ~ 4000 RBC. Once matured, gametocytes were placed in ookinete media [20] comprised of RPMI 1640, Schneider’s (Sigma-Aldrich, MO), and Waymouth’s (Mediatech, VA) medias in a 1:1:1 ratio along with 20% fetal bovine serum (FBS), 4% human RBC lysate, 0.04% NaHCO_3_ (Sigma-Aldrich, MO) 0.25% trehalose (Sigma-Aldrich, MO), adjusted to pH 7.4. The culture was incubated for 30 h in ookinete media at 27 °C with shaking at 15 RPM.

### Purification and plating of ookinetes for oocyst culture

Methods to purify *Plasmodium* ookinetes by magnetic column purification have been previously described [21]. For the studies here, mature ookinetes were incubated for 30 h and condensed into a volume of 5 mL, and then purified using LS MACS columns (Miltenyi Biotec, Bergisch Gladbach, Germany) with an attached 24 or 25-gauge flow restrictor (Strategic Applications, IL). For magnetic isolation, columns were mounted on a powerful magnet of 0.5 Tesla magnetic force (QuadroMACS Separation system; Miltenyi Biotec, Bergisch Gladbach, Germany). Prior to purification, columns were washed with RPMI. Then the culture was passed over the column while attached to the magnet. The column was washed with 10 mL of RPMI and the bound ookinetes were released by removing the column from the magnetic field and eluting in 5 mL of RPMI in a sterile collection tube. Ookinetes were washed with RPMI and resuspended in oocyst medium. Purity of the ookinetes was determined by counting ookinetes using a haemocytometer. A second round of purification using magnetic columns was performed in the same manner to obtain highly purified ookinetes. Viability of the ookinetes was determined by the Trypan blue (Amresco, OH) exclusion method. Twenty thousand purified ookinetes were seeded into individual wells of 8-well-chamber slides (Electron Microscopy Sciences, PA) that were pre-coated overnight at 4 °C with 25 μg/mL laminin (Corning, NY), 25 μg/mL laminin/entactin (Corning, NY), and/or 50 μg/mL mouse collagen IV (Corning, NY). BSA (1% BSA in PBS) was used as a negative control. Ookinetes were seeded into the pre-coated wells with oocyst medium (RPMI 1640 and Schneider’s medias in a 1:1 ratio along with 15% FBS, 0.04% w/v NaHCO_3_, 0.25% w/v trehalose, 50 μg/mL hypoxanthine (Sigma-Aldrich, MO), 10 mM HEPES (Sigma-Aldrich, MO), and 10× lipoprotein cholesterol solution [12]. Other supplements including 4% human RBC lysate, 0.015% silkworm haemolymph, and 0.001% haemin chloride (Sigma-Aldrich, MO) were added to the medium. Oocyst medium was changed on the fifth day after plating ookinetes.

### Cap380 sequence comparison across multiple *Plasmodium* species

PfCap380 protein sequences from *P. falciparum* (PFC0905c), *P. vivax* (PV095215), *P. berghei* (PB000071.00.0 and PB300510.00.0), *P. yoelii* (PY00597), and *P. gallinaceum* (PGAL8A_00395900.1) were retrieved from PlasmoDB.org. Amino acid sequence alignment was performed using ClustalW [22].

### Anti-PfCap380 antisera development

Anti-PfCap380 rabbit polyclonal antibodies (antisera) were raised by GenScript (GenScript, NJ). Briefly a gene fragment of PfCap380 corresponding to amino acids 1954–2068 (115 amino acids) was cloned into the pET-30a expression vector and expressed in BL21 (Thermo Fisher Scientific, MA) bacteria. The expressed protein contained a His-tag and was purified. The correct protein size was confirmed using SDS-PAGE, and the protein gel was stained and detained using the eSTAIN L1 staining kit (GenScript, NJ). Also, Western blot analysis was performed to identify the immunogen by presence of the His-tag. SDS-PAGE was performed and the gel was transferred to a PVDF membrane, that was washed in PBS—Tween 20 three times for 5 min each. The membrane was incubated with primary antibody (mouse-anti-His; GenScript, NJ) in milk for 30 min and washed as before. The membrane was blocked in milk and incubated with the secondary antibody (anti-mouse IgG alkaline phosphatase or HRP) for 45 min. The membrane was washed as before and then exposed to identify the immunogen, and the correct size was confirmed. Once the correct protein size and presence of the His-tag was confirmed, the purified protein immunogen was used with Freund’s adjuvant to immunize rabbits and obtain polyclonal antibodies against PfCap380.

### Immunofluorescence assays and fluorescence imaging

#### Mosquito midgut oocysts

Slightly modified methods were used for midgut immunostaining as previously described [23]. *P. falciparum* infected mosquito midguts were dissected on days 2–9, washed in cold PBS, and fixed with 4% PFA in PBS overnight at 4 °C. The next day, midguts were washed in cold PBS and blocked in blocking buffer (4% BSA in PBS) for 1 h at room temperature (RT). Midguts were permeabilized with permeabilization solution [0.05% Triton-X-100; (Sigma Aldrich), in blocking buffer] for 30 min at RT, washed thoroughly, and briefly re-blocked in blocking buffer. Midguts were incubated with anti-PfCap380 antisera (1:250 in blocking buffer) or anti-mouse-CSP antibody [24] (1:500 in blocking buffer) for 1 h at RT. The midguts were washed three times with cold PBS and blocked as previously. Midguts were incubated with secondary Alexa Fluor antibodies (1:1000 in blocking buffer) for 1 h at RT and were protected from light. All secondary antibodies were obtained from Thermo Fisher Scientific, MA and include the following: Alexa Fluor 594 goat anti-rabbit (A-11012), Alexa Fluor 594 goat anti-mouse (A-11005), Alexa Fluor 488 goat anti-rabbit (A-11008), and Alexa Fluor 647 donkey anti-mouse (A-31571). The midguts were washed as before and mounted in mounting reagent-containing 2-(4-amidinophenyl)-1H-indole-6-carboxamidine (DAPI) (Thermo Fisher Scientific, MA).

#### Gametocytes and ookinetes

Ookinetes were prepared and purified as described before, except purification was performed with one set of columns. After purification, gametocytes and ookinetes were smeared onto glass slides and fixed for 1 h using 4% PFA in PBS at RT. Slides were then washed three times using PBS and blocked overnight in blocking buffer (4% BSA in PBS) at 4 °C. The next day, the slides were permeabilized, washed, and re-blocked as described before. Slides were incubated with primary antibodies for 1–2 h at RT and washed as previously. Primary antibodies were obtained from BEI Resources (formerly MR4) and used in the following concentrations: *Pfs* 48/45 (1:500, MRA-316) and *Pfs* 230 (1:500, MRA-878A). The anti-chitinase antibody was used at 1:1000 dilution [25]. Slides were washed and re-blocked for 30 min at RT. Slides were incubated with secondary Alexa Fluor antibodies (1:1000 in blocking buffer) for 45 min at RT and were protected from light. Slides were washed as previously and then mounted as before. In some co-labelling experiments Alexa Fluors 594 and 647 were used together. During image processing, the signal of 647 channel (red) was altered to purple to contrast against the signal of the 594 channel (red) in the individual and the merged panels. IFA of ookinetes were also performed with the anti-PfCap380 antisera that was directly labelled using the Alexa Fluor 594 Antibody Labeling Kit (A20185, Thermo Fisher Scientific, MA).

#### In vitro oocysts

Immunofluorescence assays with in vitro produced oocysts were performed in 8-well-chamber slides where the oocysts were grown. After each experiment, culture media was removed and each well was washed with PBS three times and fixed with 4% PFA for 30 min at RT. Slides were washed with PBS for three times and blocked with 4% BSA in PBS for 1 h at RT. The primary antibody or antisera was diluted in blocking buffer as described before, added to each well, and incubated for 1 h at RT. Slides were washed three times with PBS and blocked as before. The slides were incubated for 1 h at RT with the secondary antibody (1:1000 in blocking buffer), washed with PBS, and then mounted in media containing DAPI.

### Microscopy

All images were taken using DeltaVision Elite High Resolution Microscope (GE Healthcare Life Science, PA) designed for fluorescence imaging and analysed using DeltaVision software (SoftWoRx software version 6.5.2). Images were taken at 40×, 60×, or 100× magnification with a 10× eyepiece. Merging of separate colour channels was performed using Image J [26].

### Early oocyst binding assay and counting of early oocysts

Eight-well-chamber slides were pre-coated over night with laminin, collagen type IV, and entactin, as described. BSA was used as a negative control. After coating, excess material was removed and washed with sterile PBS. Fixed numbers of ookinetes (20,000 per well) were seeded and allowed to transform into early oocysts after 2, 3, and 6 days in culture. At the end of 2, 3, or 6 days completing the developmental experiment, the wells were washed, fixed and stained as described before with anti-PfCap380 antisera. For each condition, a total of 20 fields (containing ~ 700 oocysts) were counted at 40× magnification. For each experiment, average values of oocyst binding per field were determined and then normalized to BSA. Each group was compared to BSA and statistical significance was determined using Student’s T tests.

### DNA quantification from in vitro oocysts

To quantify DNA from oocysts, ookinetes were seeded into 6-well-plates, and oocysts were transformed and grown as described above using laminin, entactin, and collagen IV as the basal lamina. On days 2, 3, and 6, oocysts were collected in PBS to count the number of live oocysts. Oocysts were resuspended in 400 μL of lysis buffer (0.2% Triton in Tris–EDTA buffer). Total double stranded DNA (dsDNA) from 12,500 live oocysts was measured per sample using the Quant-iT PicoGreen dsDNA Assay Kit according to the manufacturer’s protocol. (P7589, Thermo Fisher Scientific, MA). To each oocyst sample, 100 μL of PicoGreen solution was added. Then, oocysts were gently shaken and incubated for 5 min before measuring fluorescence using a microplate reader (SpectraMax M2, Molecular Devices, CA) at wavelengths 480 for excitation and 520 for emission. Total oocyst DNA concentration was calculated using a known DNA standard.

## Results

### Cap380 is an oocyst-specific marker

To identify a stage-specific oocyst marker for oocyst developmental studies, a thorough literature search was performed. The only known oocyst-specific protein to date is an oocyst capsule protein, Cap380, which was originally identified in *P. berghei* (PbCap380) from a subtraction library of genes enriched for expression during the oocyst stage [27]. Cap380 is a unique protein and all *Plasmodium* species have Cap380 orthologues. When comparing the Cap380 amino acid sequence across several *Plasmodium* species, the N-terminal half of the protein shows greater similarity than the C-terminal half [13]. In order to select the PfCap380 peptide immunogen, the region of *P. falciparum* that is homologous to the antigenic region of *P. berghei* was analyzed [13]. From these amino acids in *P. falciparum* (1954–2283), a shorter peptide fragment (1954–2068) within the larger region was selected since the expressed peptide was predicted to exhibit high stability. Next, this region of *Pf*Cap380 was compared to the orthologues for *P. berghei*, *P. yoelii, P. vivax,* and *P. gallinaceum* and it was found that PfCap380 shares greatest amino acid similarity with *P. gallinaceum* followed by *P. vivax, P. berghei* and *P. yoelii* (see Additional file 1: Figure S1A, B). The general dissimilarity of amino acids between *P. falciparum* and *P. berghei* in this region explains why the anti-PbCap380 antibody that was previously generated does not cross react with *P. falciparum* (unpublished observations). PfCap380 was selected as an immunogen for antibody design since it is expressed in the oocyst stage and exhibits stage-specific expression [13].

### Immunogen design and generating anti-PfCap380 antisera

While a previous study in *P. berghei* generated an antibody against PbCap380, the anti-PbCap380 antibody does not cross-react with *P. falciparum* infected midgut oocysts, prohibiting usability in *P. falciparum* oocyst developmental studies [13], Unpublished observations]. Therefore, antisera against PfCap380 was raised using a similar strategy that was used for the anti-PbCap380 antibody [13]. The first 115 amino acids of PfCap380 (1954–2068) homologous to the peptide fragment of PbCap380 that was used for immunization was selected. The expressed His-tagged PfCap380 peptide fragment showed the predicted size in SDS-PAGE analysis, ~ 15 kDa (See Additional file 2: Figure S2A, performed by GenScript). Western blot analysis using an anti-His antibody also confirmed expression of the same size protein fragment (See Additional file 2: Figure S2B, performed by GenScript). The PfCap380 peptide immunogen was then used to raise polyclonal rabbit antibodies by using one prime immunization followed by two boosting immunizations. In sum, an immunogen corresponding to PfCap380 amino acids 1954–2068 was used to immunize rabbits and obtain polyclonal antisera for *P. falciparum* oocyst developmental studies.

### PfCap380 expression begins in early midgut oocysts

**Fig. 1:**
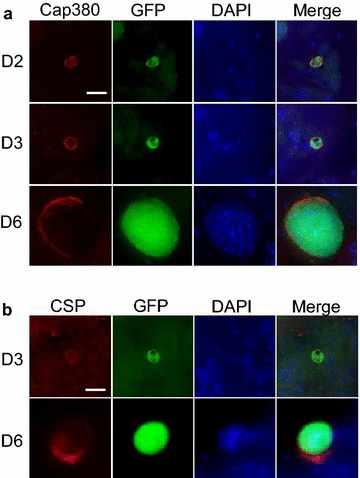
Expression of PfCap380 and CSP in mosquito derived oocysts. IFA were performed on GFP-Luc infected midguts at the indicated time points (*D* Day). **a** Midguts oocysts were labeled with primary antisera (red) against PfCap380 (**a**) or CSP (**b**), and show expression of GFP in green and nuclear staining with DAPI in blue. The merged image of the three separate channels is shown in the last panel. Scale bars = 10 μm

**Fig. 2:**
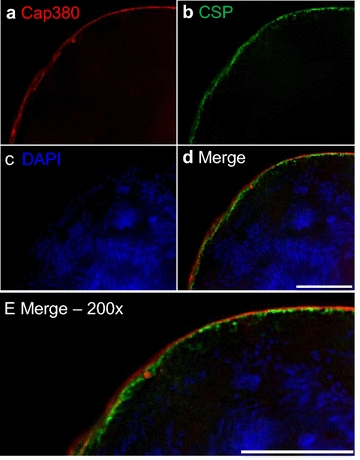
Expression of PfCap380 and colocalization of CSP in mosquito derived oocysts. Day 9 infected midgut oocysts were labelled with primary antibodies against PfCap380 in red (**a**) and CSP in green (**b**) and with DAPI in blue to stain oocyst DNA (**c**). The merged image of the three channels shows the stained capsule and multi-nucleated oocyst (**d**). The enlargement in **e** shows the localization of PfCap380 and CSP. CSP appears to be localized on the plasma membrane below the oocyst capsule containing PfCap380 (**d**). Scale bars = 10 μm

Towards developing a culturing system for production of *P. falciparum* mosquito stages, including oocysts, in vivo oocyst development was first studied to serve as a standard. Oocysts were identified in mosquito midguts by following GFP expression using a fluorescent reporter strain, *P. falciparum* NF54HT-GFP-luc [14]. Oocysts were generally round or oval in shape and grew in size during the course of the developmental study (from 2 to 9 days after mosquito infection). The measured oocyst diameter was ~ 7 μm on days 2–3, ~ 20 μm on day 6, and ~ 30–40 μm on day 9. To study in vivo oocyst development of *P. falciparum* infected mosquito midgut oocysts, the expression of PfCap380 was monitored by immunofluorescence assays (IFA) using anti-PfCap380 antisera. The expression of PfCap380 was found to begin on day 2 as a small circular or oval pattern surrounding the oocysts and was present at all time points (days 2–9) (Figs. 1a and 2). No signal was observed for negative controls using only secondary antibodies (See Additional file 3: Figure S3A). These results demonstrate that midgut *P. falciparum* oocysts express Cap380, and that Cap380 can be recognized by the anti-PfCap380 antisera.

Circumsporozoite protein (CSP) is another oocyst marker, but is expressed in other stages than the oocyst since it is also expressed in SPZ. Previous studies using fluorescence and immunoelectron microscopy demonstrate that CSP is localized on the oocyst plasma membrane [13, 28–30] directly below the oocyst capsule where Cap380 is localized [13]. Separate *P. falciparum* developmental studies on in vivo oocyst expression of CSP using western blot and ELISA methods found CSP to be expressed on days 8–10 after mosquito infection [31, 32], while immunoelectron microscopy of *P. berghei* oocysts demonstrate low but detectable CSP expression in early oocysts [28]. IFA were performed on *P. falciparum* mosquito-derived oocysts to study CSP and compare the utility of CSP to Cap380 as an oocyst marker. In contrast to the PfCap380 expression patterns, CSP was expressed later and in a different outer layer of the oocyst. CSP signal was not found on day 2 as it was for Cap380. Instead, CSP expression was very low but detectable on day 3 and was higher on days 6 and 9 (Figs. 1b, 2). This high-resolution fluorescence microscopy study revealed multi-nucleated oocyst DNA below the oocyst membrane and capsule using DAPI staining (Fig. 2c–e). The data also suggest that CSP appears to localize below PfCap380 (Fig. 2e) but higher resolution microscopy (immunoelectron microscopy) would be required to be absolutely certain the two proteins are located separately. Other studies demonstrate that CSP localizes to the plasma membrane [28–30] directly below the oocyst capsule, where PbCap380 is localized [13]. The present studies demonstrate the utility of PfCap380 as a marker and the anti-PfCap380 antisera to study in vivo oocyst development from days 2–9 after mosquito infection.

### The basal lamina component, collagen IV supports robust binding of early oocysts

**Fig. 3:**
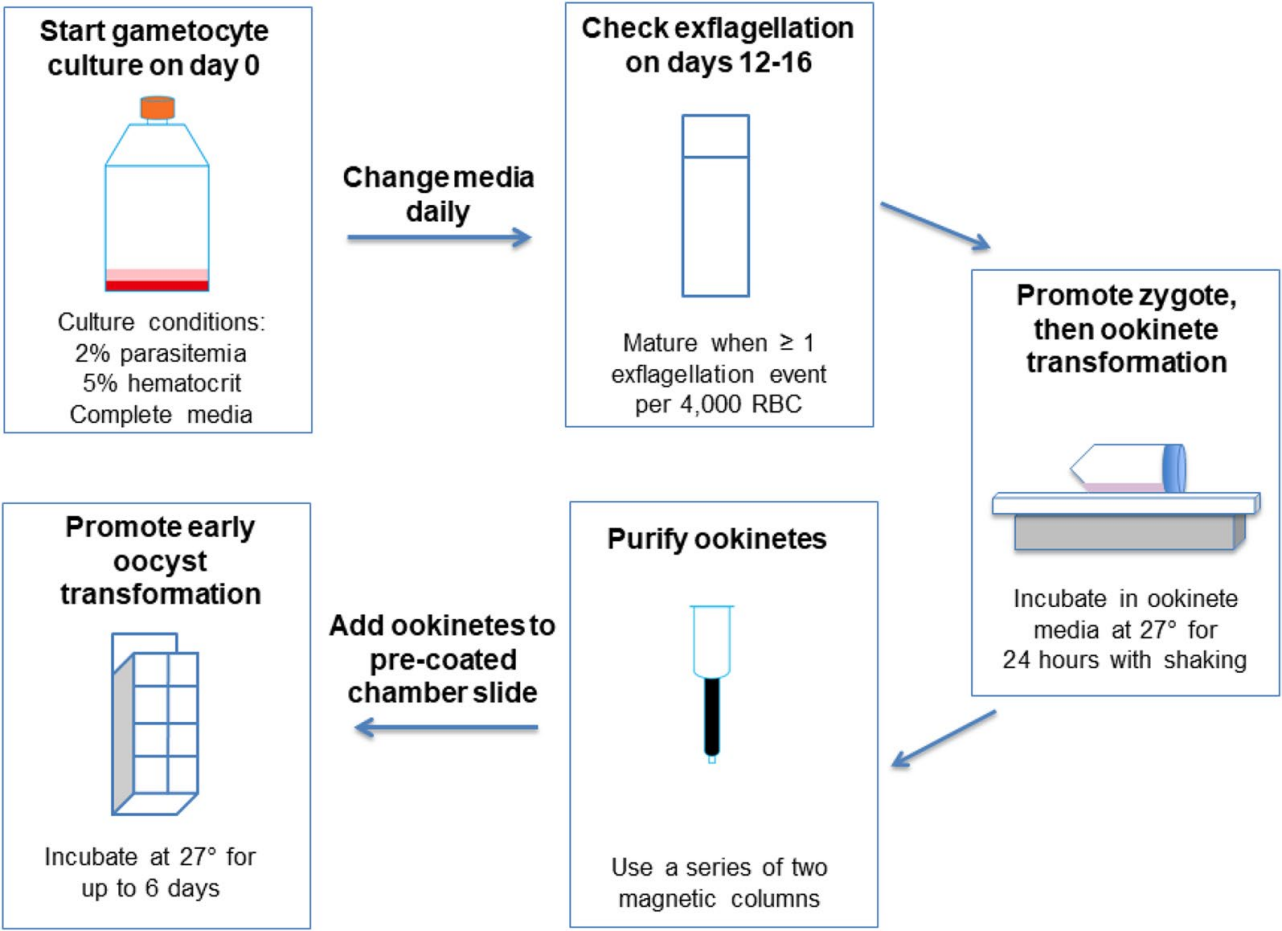
Flow diagram of in vitro oocyst culture. Gametocyte culture was seeded from an asexual culture at 2% parasitaemia and 5% hematocrit in 30 mL of complete media (RPMI with 10% A + human serum). Two flasks of 30 mL cultures were used for each experiment. Media changes were performed daily and percent gametocytaemia was determined. When one or more exflagellation events in 4000 RBC was reached, the culture was considered mature. Once the culture was matured, two flasks were combined, pelleted, and resuspended in 5 mL of ookinete media. Zygotes formed after 5 h with incubation at 27 °C and shaking at 50 RPM. Ookinetes transformed after a total of 24 h of incubation at 27 °C and shaking at 15 RPM. Ookinetes were purified using two sequential magnetic columns. Purified ookinetes were resuspended in oocyst media and plated into 8-well-chamber slides that were pre-coated with basal lamina components. Oocysts were incubated at 27 °C for up to 6 days in oocyst media

A goal of this work is to study *P. falciparum* mosquito stage development in vitro to ultimately manufacture SPZ for malaria vaccines. In vitro oocysts were produced by culturing *P. falciparum* gametocytes to maturity, then promoting the parasites to transform through the stages—zygote, ookinete, and oocyst—as outlined in Fig. 3. Zygotes and ookinetes were produced by making minor modifications to an existing protocol [20, 33]. Once ookinetes formed, magnetic column purification was used to remove red blood cells [21] and obtain highly purified ookinetes. For *Plasmodium* development in the mosquito, ookinetes bind to the mosquito midgut basal lamina, and this interaction is thought to trigger the transformation of ookinetes into early oocysts [34]. Considering this knowledge, basal lamina components were provided along with nutrient rich media (oocyst media) to mature ookinetes, supporting transformation into early oocysts. Then, oocysts were studied over a 6-day developmental time course.

**Fig. 4:**
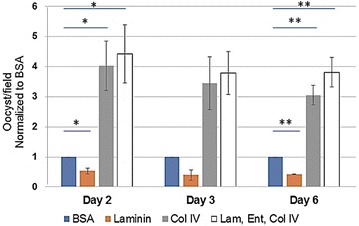
Binding affinity of early oocysts to basal lamina components. Several basal lamina components were tested for oocyst binding, including laminin, collagen IV, or the combination of laminin, entactin, and collagen IV (Lam, Ent, Col IV). BSA was used as a negative control. The number of oocysts per field were counted and shown as fold change values compared to BSA. The average number is shown for three experiments on days 2 and 6 and for two experiments on day 3. Error bars represent standard deviation. Student’s T Tests were performed to compare each group to BSA, and * = p < 0.05, ** = p < 0.01

To select basal lamina components that would support ookinete binding and transformation into oocysts, *Plasmodium* development in mosquitoes was considered as well as the previously published *P. falciparum* culturing system. The study by Warburg and Schneider utilized Matrigel as a basal lamina, which is a gel secreted by Engelbreth-Holm-Swarm mouse sarcoma cells and contains proteins including laminin, entactin, and collagen IV [12]. Matrigel also contains growth factors in undefined amounts and would be problematic for use in manufacturing human vaccines due to lack of complete characterization of unknown, tumor derived factors. Therefore, this study was aimed to identify the minimal basal lamina requirements for in vitro oocyst production and focused on the protein components of Matrigel. Laminin and collagen IV are known components of the mosquito basal lamina and are considered important for ookinete binding and parasite development [34–38]. Laminin, a glycoprotein, incorporates into the developing oocyst [39] while collagen IV is a structural sheet-forming protein of the basal lamina. Entactin is a glycoprotein that connects laminin and collagen IV. The tested basal lamina components include laminin alone, collagen IV alone, and a combination of laminin, entactin and collagen IV (L/E/C). The number of oocysts per field was counted and shown as fold change compared to BSA (negative control) (Fig. 4). On day 2, fold change values were as follows, laminin—0.53, collagen IV—4.0, L/E/C—4.4; on day 3, laminin—0.4, collagen IV—3.4, L/E/C—3.8; on day 6, laminin—0.4, collagen IV—3.0, L/E/C—3.8. The binding study of *P. falciparum* to basal lamina components revealed that collagen IV alone or in combination with laminin and entactin provided the greatest binding compared to controls (BSA) or laminin alone.

High transformation rates for gametocytes to ookinetes (24 ± 0.02%) and ookinetes to early oocysts (85 ± 0.04%) was observed using the L/E/C basal lamina (See Additional file 4: Figure S4). Within 24 h of in vitro development, ookinetes round up to form early oocysts, attach to the reconstituted basal lamina, and begin growing. The oocyst media consists of components described by Warburg and Schneider [12], but without the use of *Drosophila* S2 cells. Novel supplements to the oocyst media include human RBC lysate, silkworm hemolymph, and hemin chloride. After screening several factors, the novel supplements were found to support oocyst transformation and viability. Early oocysts grew in size from day 2 to 6 reaching the maximum size ~ 5–7 μm; however, most of the oocysts were developmentally arrested, but still viable (positive GFP signal) for ~ 7 more days. In sum, the oocyst culturing system promotes high ookinete to early oocyst transformation by using collagen as a basal lamina component and novel oocyst media.

### In vitro oocysts express PfCap380 from the second day after transformation

**Fig. 5:**
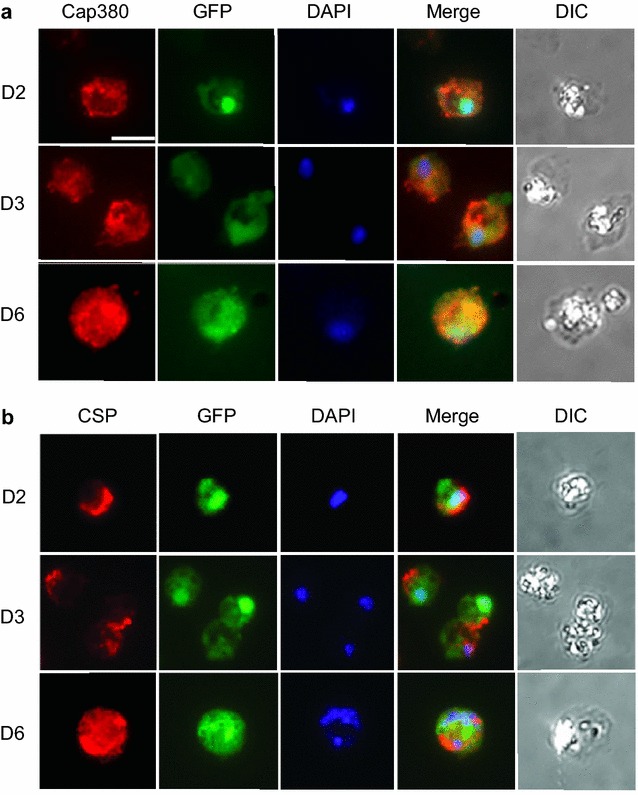
Expression of PfCap380 in early in vitro produced oocysts. IFA were performed on in vitro oocysts at the indicated time points, (*D* Day). Oocysts were labeled with primary antisera (red) against PfCap380 (**a**) or CSP (**b**), showing expression of GFP in green and DAPI nuclear staining in blue. The merged image of the three separate channels is shown as well as the corresponding DIC image. Scale bars = 5 μm

To study the development of in vitro produced oocysts, the expression and localization of PfCap380 and CSP was studied by IFA on days 2, 3, and 6 after seeding ookinetes (Fig. 5). Similar to mosquito-derived oocysts, in vitro oocysts expressed PfCap380 from day 2 and maintained the expression pattern up to day 6, (Fig. 5a). The majority of oocysts (> 90%) exhibited a similar expression pattern to the images shown (Fig. 5). On day 6, most oocysts showed strong PfCap380 staining as compared to the negative controls (See Additional file 3: Figure S3B). For CSP, expression began at day 2 and continued through day 6. However, in most oocysts, only a portion of the oocyst circumference showed CSP staining (Fig. 5b).

**Fig. 6:**
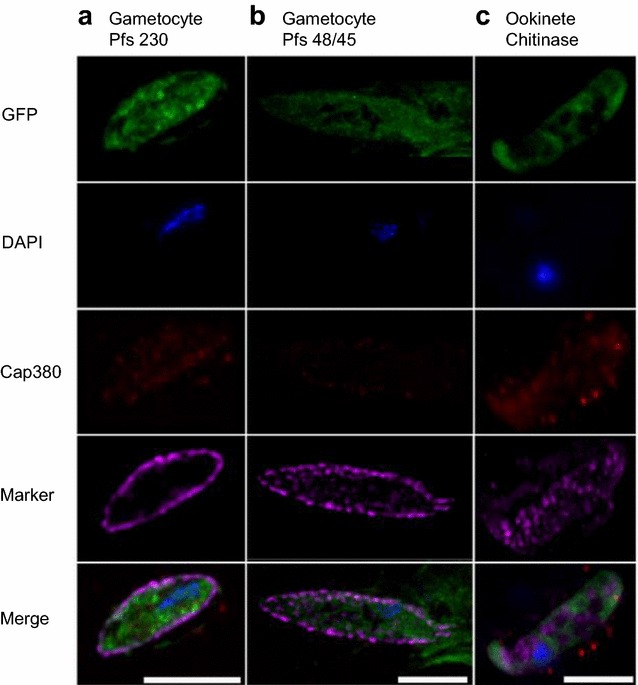
Gametocytes do not express PfCap380. Purified GFP-Luc gametocytes and ookinetes were labelled with anti-PfCap380 antisera and co-labeled with antibodies against gametocyte (P*f*s230, P*f*s48/45) or ookinete (chitinase) markers. Gametocytes and ookinetes express GFP in green and nuclei stain with DAPI in blue. PfCap380 is shown in red; Pfs230 (**a**), Pfs48/45 (**b**), and chitinase (**c**) are in purple. The merged image of the four separate channels is shown in the bottom row. Gametocytes express Pfs230 and P*f*s48/45 but not PfCap380. Ookinetes express chitinase but not PfCap380. Scale bars = 10 μm in **a**; 7.5 μm in **b** and **c**

To test whether PfCap380 is expressed only in the oocyst stage or also in other mosquito stages, IFA were performed on gametocytes and ookinetes using anti-PfCap380 and co-labeled using antibodies against gametocyte markers (anti-Pfs230 and anti-Pfs48/45) and an ookinete marker (anti-chitinase). PfCap380 is not expressed in gametocytes or ookinetes; although, the background staining for PfCap380 was greater for ookinetes than for gametocytes (Fig. 6). To confirm this was background or non-specific staining rather than specific staining, the anti-PfCap380 antisera was directly labelled with a fluorophore and under these conditions, anti-PfCap380 does not bind ookinetes (See Additional file 5: Figure S5). An earlier study of PbCap380 expression also showed that Cap380 expression is restricted to the oocyst stage [13]. Since the IFA data showed binding of the PfCap380 antisera only to oocyst stages as previously reported for *P. berghei*, additional control experiments with the antisera were not performed (i.e., SDS-page or Western blot analysis on parasite extracts or mosquitoes). All gametocytes express Pfs230, while only a subset of gametocytes express Pfs48/45, and ookinetes express chitinase (Fig. 6). The observed expression patterns for gametocyte and ookinete markers are also in agreement with previous studies [40, 41]. The IFA studies on in vitro produced *P. falciparum* support oocyst expression of PfCap380 and utility of anti-PfCap380 antisera on in vitro produced oocysts.

### In vitro oocysts undergo DNA replication

A previous study using the *P. berghei* in vitro culturing system demonstrates that oocyst endomitosis occurs by showing that DAPI foci increase over a 15-day time course [10]. To determine whether genome replication occurs in the present in vitro culturing system, total double-stranded DNA (dsDNA) was measured using PicoGreen, which binds specifically to dsDNA. The average concentration values (ng/mL) were as follows for day 2–16.3; day 3–19.4, and day 6–23.9 (See Additional file 6: Figure S6). These data indicate that DNA replication occurs during the six-day time course of the in vitro oocyst culture.

## Discussion

### The capsule and Cap380 on in vivo oocysts

The oocyst capsule begins forming soon after the transition from ookinetes to early oocysts and increases in size to accommodate several thousands of growing SPZ. The actual mechanism of how the oocyst capsule forms and grows is not completely understood, yet it is believed that the capsule is an ordered and dynamic structure that facilitates transport of nutrients into the oocyst and exit of metabolites [42]. The capsule is not a lipid bilayer membrane, and is presumed to consist of several layers of proteins and carbohydrate moieties. Studies support a role for the capsule in protecting developing SPZ from the mosquito immune system [13, 43].

The localization of Cap380 begins on the surface of early oocysts and incorporates into the oocyst capsule [13]. Previous microarray studies on *P. berghei* showed that PbCap380 is expressed in oocysts but not in gametocytes, SPZ, nor in blood stage parasites [44]. In the present studies, Cap380 localizes to the capsule of *P. falciparum* oocysts, and is not expressed in other sexual stages (gametocytes or ookinetes) (Fig. 6, See Additional file 5: Figure S5). The microscopy study presented suggests that the *P. falciparum* oocyst capsule consists of Cap380, and CSP appears to underlie Cap380 on the oocyst plasma membrane (Fig. 2). While the exact function of Cap380 is not yet known, knockout studies of PbCap380 indicate the importance of the protein for oocyst survival and SPZ production. In these studies, normal numbers of early oocysts formed, but further development was blocked and oocysts were eliminated without production of SPZ [13].

The anti-PfCap380 antisera presented here binds to the midgut oocyst capsule from day 2 and through day 9 (Fig. 2). While CSP can be used to study oocyst development, strong CSP expression is not seen until 6 days after mosquito infection, and CSP is not a stage-specific marker as it is also strongly expressed in SPZ. To study oocyst development in the infected mosquito midgut, anti-PfCap380 antisera serves as a powerful stage-specific reagent to monitor oocyst growth and development as early as 2 days after infection with gametocytes.

### In vitro transformed oocysts express *Pf*Cap380

Ookinetes bind to the mosquito midgut basal lamina, and this interaction is thought to trigger the transformation of ookinetes into early oocysts [34]. In the present study, basal lamina components were tested for binding affinity of early oocysts and, therefore, ability to support transformation into early oocysts. The results demonstrate the need for basal lamina components, especially collagen IV, since collagen IV alone or together with laminin and entactin support early oocyst transformation when compared to controls or laminin alone (Fig. 4). A high rate of ookinete to oocyst transformation (85%) was observed. The only other published study on in vitro production of *P. falciparum* oocysts did not describe the oocyst transformation rate [12]. The high rate of transformation in the present culturing system could be due to high viability of ookinetes (> 95%) or possibly to culturing in the absence of the mosquito immune response, which counteracts parasite survival.

Early in vitro *P. falciparum* oocysts produced in the present culturing system exhibit the following characteristics: (1) Transformation of ookinetes into oocysts (i.e., rounding up into oocysts) within 24 h; and (2) Development to the diameter of ~ 5–7 μm. These results are consistent with other early oocyst *Plasmodium* culturing systems [9, 10, 45]. The anti-PfCap380 antisera detects early oocysts from day 2 and the staining pattern is distributed on the entire surface of the oocyst capsule (Fig. 5a). CSP expression occurs later as consistently strong expression was observed starting on day 6 (Fig. 5b). The in vitro development of oocysts arrests after day 6, and DNA replication occurs as a significant but slow increase in DNA concentration was observed. These results can be explained by nutritional deficiency in the oocyst media or lack of proper signaling molecules for further oocyst maturation. Additional studies on an improved, more robust in vitro culturing system will be necessary for production of mature SPZ-producing oocysts.

The present study demonstrates the utility of PfCap380 as a marker to study in vitro oocyst development. The anti-PfCap380 antisera will serve as a powerful reagent for screening optimal basal lamina components, growth factors, and nutritional supplements for the oocyst culturing system to promote the optimal transformation rates and maturation of in vitro oocysts. Production of large quantities of early and mature oocysts in a culturing system will enable—omics level studies on oocysts, completing knowledge gaps in the least studied *Plasmodium* life cycle stage—oocysts. Importantly, the subsequent development of SPZ from mature oocysts in vitro will support scalable manufacturing of whole SPZ for malaria vaccines.

## Conclusions

These studies present in vitro methods to culture mosquito stage equivalents of the *P. falciparum* parasite (i.e., gametes, zygotes, ookinetes and early oocysts), and antisera against the oocyst capsule protein, PfCap380 for oocyst studies. The antisera enabled developmental studies of oocysts derived from mosquitoes (in vivo) and from culture (in vitro). PfCap380 expression began on early in vivo oocysts (day 2) and continued to day 9. For in vitro oocysts, PfCap380 was expressed from day 2 to day 6 and arrested growth compared to in vivo oocysts. Necessary basal lamina components were identified for ookinete to oocyst transformation in vitro. Future studies will focus on promoting in vitro oocyst development to produce SPZ for malaria vaccines. An in vitro platform to manufacture SPZ should enable the production of any *P. falciparum* SPZ type, including GAP or WT, which could be irradiated (RAS) or administered along with chemoprophylaxis (CPS) for use in mass production of a malaria vaccine.

## Additional files

**Additional file 1: Figure S1.** Sequence alignment of *Plasmodium* Cap380 orthologues. Amino acid sequence alignment of the PfCap380 proteins for several *Plasmodium* species was performed to identify similarities within the peptide immunogen region. In A, Asterisks = fully conserved residues, colon = conservation with strongly similar properties, period = conservation with weakly similar properties. In B, the percentages of similar, identical, or gap amino acids are shown for the PfCap380 antigen compared to *Plasmodium* species. PfCap380 shares most amino acid similarity with *Pg*, followed by *Pv, Pb*, and then *Py*.

**Additional file 2: Figure S2.** Expression of the PfCap380 peptide antigen. An image of an SDS-PAGE protein gel shows the migration of the purified His-tagged-PfCap380 peptide band in A (arrow). Western blot analysis shows the same peptide fragment recognized by an anti-His antibody in panel B (arrow). Protein molecular weight markers (10-120 or 22-120 kDa) are indicated. These results were generated by GenScript and shown with their permission.

**Additional file 3: Figure S3.** Negative control IFA on *in vivo* and *in vitro* oocysts. IFA were performed as described except no primary antisera was used to test secondary antibodies for non-specific binding. Secondary antibodies were used to label midgut oocysts (A) or *in vitro* oocysts (B) and show expression of GFP in green and DAPI nuclear staining in blue. The merged image of the three separate channels is shown. DIC images were taken for *in vitro* but not *in vivo* oocysts due to challenges in imaging midgut tissue. Scale bars = 10 μm. For midgut oocysts, negative control antibodies used were Alexa Fluor 594 anti-rabbit for Cap380 and Alexa Fluor 647 anti-mouse for CSP. For *in vitro* oocysts, negative control antibodies used were Alexa Fluor 594 anti-rabbit for Cap380 and Alexa Fluor 594 anti-mouse for CSP.

**Additional file 4: Figure S4.** Transformation rates between parasite stages. The graph shows the transformation rates for gametocyte to ookinete stages and ookinete to early oocyst stages. The gametocyte to ookinete transformation rate was determined by counting gametocytes and ookinetes in a hemocytometer. The ookinete to oocyst transformation rate was determined by counting oocysts in an 8-well-chamber slide that formed after seeding a known quantity of ookinetes. The values depict averages across three experiments and error bars represent standard deviation.

**Additional file 5: Figure S5.** Ookinetes do not express PfCap380. IFA were performed using purified ookinetes with anti-PfCap380 antisera directly labeled with Alexa Fluor 594. Ookinetes (1-4) express GFP in green and nuclei stain with DAPI in blue but do not express PfCap380 (red). The merged image of the four separate channels is shown. Scale bar = 5 μm.

**Additional file 6: Figure S6.** Oocyst DNA increases after six days of *in vitro* culture. After two, three, and six days in culture, oocysts were collected and DNA content was measured using a fluorescent nucleic acid stain specific for double-stranded DNA (dsDNA). dsDNA concentration was calculated by comparison to standards with known DNA concentrations. Mean values are shown for oocysts on days 2, 3 and 6. To determine the significance between groups, a One-way ANOVA and Tukey’s Test was performed. For significance, * = p < 0.05, ** = p < 0.01. The experiment was performed in triplicate, and error bars represent standard deviation.

## Abbreviations

CSP: circumsporozoite protein
Cap: capsule protein
SPZ: sporozoites
GAP: genetically attenuated parasites
RAS: radiation attenuated sporozoites
CPS: chloroquine chemoprophylaxis with sporozoites

## References

[1] WHO (2015). World Malaria report 2015.

[2] RTS, S Clinical Trials Partnership (2015). Efficacy and safety of RTS, S/AS01 malaria vaccine with or without a booster dose in infants and children in Africa: final results of a phase 3, individually randomised, controlled trial. Lancet.

[3] Mikolajczak SA, Lakshmanan V, Fishbaugher M, Camargo N, Harupa A, Kaushansky A (2014). A next-generation genetically attenuated *Plasmodium falciparum* parasite created by triple gene deletion. Mol Ther.

[4] Hoffman SL, Vekemans J, Richie TL, Duffy PE (2015). The march toward malaria vaccines. Vaccine.

[5] Mordmüller B, Surat G, Lagler H, Chakravarty S, Ishizuka AS, Lalremruata A (2017). Sterile protection against human malaria by chemoattenuated PfSPZ vaccine. Nature.

[6] Kublin JG, Sack BK, Fishbaugher ME, Seilie A, Shelton L, VonGoedert T (2017). Complete attenuation of genetically engineered *Plasmodium falciparum* sporozoites in human subjects. Sci Transl Med.

[7] Labaied M, Harupa A, Dumpit RF, Coppens I, Mikolajczak SA, Kappe SH (2007). *Plasmodium yoelii* sporozoites with simultaneous deletion of P52 and P36 are completely attenuated and confer sterile immunity against infection. Infect Immun.

[8] van Schaijk BC, Ploemen IH, Annoura T, Vos MW, Foquet L, van Gemert GJ (2014). A genetically attenuated malaria vaccine candidate based on *P. falciparum* b9/slarp gene-deficient sporozoites. Elife.

[9] Warburg A, Miller LH (1992). Sporogonic development of a malaria parasite in vitro. Science.

[10] Al-Olayan EM, Beetsma AL, Butcher GA, Sinden RE, Hurd H (2002). Complete development of mosquito phases of the malaria parasite in vitro. Science.

[11] Porter-Kelley JM, Dinglasan RR, Alam U, Ndeta GA, Sacci JB, Azad AF (2006). *Plasmodium yoelii*: axenic development of the parasite mosquito stages. Exp Parasitol.

[12] Warburg A, Schneider I (1993). In vitro culture of the mosquito stages of *Plasmodium falciparum*. Exp Parasitol.

[13] Srinivasan P, Fujioka H, Jacobs-Lorena M (2008). PbCap380, a novel oocyst capsule protein, is essential for malaria parasite survival in the mosquito. Cell Microbiol.

[14] Vaughan AM, Mikolajczak SA, Camargo N, Lakshmanan V, Kennedy M, Lindner SE (2012). A transgenic *Plasmodium falciparum* NF54 strain that expresses GFP-luciferase throughout the parasite life cycle. Mol Biochem Parasitol.

[15] Haynes DJ, Diggs CL, Hines FA, Desjardins RE (1976). Culture of human malaria parasites *Plasmodium falciparum*. Nature.

[16] Trager W, Jensen JB (1976). Human malaria parasites in continuous culture. Science.

[17] Ifediba T, Vanderberg JP (1981). Complete in vitro maturation of *Plasmodium falciparum* gametocytes. Nature.

[18] Ponnudurai T, Meuwissen JH, Leeuwenberg AD, Verhave JP, Lensen AH (1982). The production of mature gametocytes of *Plasmodium falciparum* in continuous cultures of different isolates infective to mosquitoes. Trans R Soc Trop Med Hyg.

[19] Vaughan AM, Mikolajczak SA, Wilson EM, Grompe M, Kaushansky A, Camargo N (2012). Complete *Plasmodium falciparum* liver-stage development in liver-chimeric mice. J Clin Invest.

[20] Ghosh AK, Dinglasan RR, Ikadai H, Jacobs-Lorena M (2010). An improved method for the in vitro differentiation of *Plasmodium falciparum* gametocytes into ookinetes. Malar J.

[21] Carter V, Cable HC, Underhill BA, Williams J, Hurd H (2003). Isolation of *Plasmodium berghei* ookinetes in culture using Nycodenz density gradient columns and magnetic isolation. Malar J.

[22] Higgins DG, Sharp PM (1988). CLUSTAL: a package for performing multiple sequence alignment on a microcomputer. Gene.

[23] Han YS, Thompson J, Kafatos FC, Barillas-Mury C (2000). Molecular interactions between *Anopheles stephensi* midgut cells and *Plasmodium berghei*: the time bomb theory of ookinete invasion of mosquitoes. EMBO J.

[24] Sack BK, Miller JL, Vaughan AM, Douglass A, Kaushansky A, Mikolajczak S (2014). Model for in vivo assessment of humoral protection against malaria sporozoite challenge by passive transfer of monoclonal antibodies and immune serum. Infect Immun.

[25] Vinetz JM, Dave SK, Specht CA, Brameld KA, Xu B, Hayward R (1999). The chitinase PfCHT1 from the human malaria parasite *Plasmodium falciparum* lacks proenzyme and chitin-binding domains and dislays unique substrate preferences. Proc Natl Acad Sci USA.

[26] Schneider CA, Rasband WS, Eliceiri KW (2012). NIH Image to ImageJ: 25 years of image analysis. Nat Methods.

[27] Srinivasan P, Abraham EG, Ghosh AK, Valenzuela J, Ribeiro JM, Dimopoulos G (2004). Analysis of the *Plasmodium* and *Anopheles* transcriptomes during oocyst differentiation. J Biol Chem.

[28] Thathy V, Fujioka H, Gantt S, Nussenzweig R, Nussenzweig V, Menard R (2002). Levels of circumsporozoite protein in the Plasmodium oocyst determine sporozoite morphology. EMBO J.

[29] Posthuma G, Meis JF, Verhave JP, Hollingdale MR, Ponnudurai T, Meuwissen JH (1988). Immunogold localization of circumsporozoite protein of the malaria parasite *Plasmodium falciparum* during sporogny in *Anopheles stephensi* midguts. Eur J Cell Biol.

[30] Golenda CF, Starkweather WH, Wirtz RA (1990). The distribution of circumsporozoite protein (CS) in *Anopheles stephensi* mosquitoes infected with *Plasmodium falciparum* malaria. J Histochem Cytochem.

[31] Kumar S, Zheng H, Deng B, Mahajan B, Grabias B, Kozakai Y (2014). A slot blot immunoassay for quantitative detection of *Plasmodium falciparum* circumsporozoite protein in mosquito midgut oocyst. PLoS ONE.

[32] Stone W, Grabias B, Lanke K, Zheng H, Locke E, Diallo D (2015). A comparison of *Plasmodium falciparum* circumsporozoite protein-based slot blot and ELISA immuno-assays for oocyst detection in mosquito homogenates. Malar J.

[33] Ghosh AK, Jacobs-Lorena M (2013). In vitro differentiation of *Plasmodium falciparum* gametocytes into ookinetes. Methods Mol Biol.

[34] Arrighi RB, Hurd H (2002). The role of *Plasmodium berghei* ookinete proteins in binding to basal lamina components and transformation into oocysts. Int J Parasitol.

[35] Adini A, Warburg A (1999). Interaction of *Plasmodium gallinaceum* ookinetes and oocysts with extracellular matrix proteins. Parasitology.

[36] Vlachou D, Lycett G, Siden-Kiamos I, Blass C, Sinden RE, Louis C (2001). *Anopheles gambiae* laminin interacts with the P25 surface protein of *Plasmodium berghei* ookinetes. Mol Biochem Parasitol.

[37] Dessens JT, Sinden-Kiamos I, Mendoza J, Mahairaki V, Khater E, Vlachou D (2003). SOAP, a novel malaria ookinete protein involved in mosquito midgut invasion and oocyst development. Mol Microbiol.

[38] Gare DC, Piertney SB, Billingsley PF (2003). *Anopheles gambiae* collagen IV genes: cloning, phylogeny, and midgut expression associated with blood feeding and Plasmodium infection. Int J Parasitol.

[39] Nacer AM, Walker K, Hurd H (2008). Localisation of laminin within *Plasmodium berghei* oocysts and the midgut epithelial cells of *Anopheles stephensi*. Parasit Vectors.

[40] Quakyi IA, Carter R, Rener J, Kumar N, Good MF, Miller LH (1987). The 230-kDa gamete surface protein of *Plasmodium falciparum* is also a target for transmission-blocking antibodies. J Immunol.

[41] Rener J, Graves PM, Carter R, Williams JL, Burkot TR (1983). Target antigens of transmission-blocking immunity on gametes of *Plasmodium falciparum*. J Exp Med.

[42] Vanderberg J, Rhodin J (1967). Differentiation of nuclear and cytoplasmic fine structure during sporogonic development of *Plasmodium berghei*. J Cell Biol.

[43] Pringle G (1996). A quantitative study of naturally-acquired malaria infections in *Anopheles gambiae* and *Anopheles funestus* in a highly malarious area of East Africa. Trans R Soc Trop Med Hyg.

[44] Le Roch KG, Zhou Y, Blair PL, Grainger M, Moch JK, Haynes JD (2003). Discovery of gene function by expression profiling of the malaria parasite life cycle. Science.

[45] Carter V, Nacer AM, Underhill A, Sinden RE, Hurd H (2007). Minimum requirements for ookinete to oocyst transformation in Plasmodium. Int J Parasitol.

