# Supplementary figures and images for: PfCap380 as a marker for Plasmodium falciparum oocyst development in vivo and in vitro
Source: Malar J. 2018 Apr 2;17:135. doi: 10.1186/s12936-018-2277-6 (PMC5880026; doi:10.1186/s12936-018-2277-6)

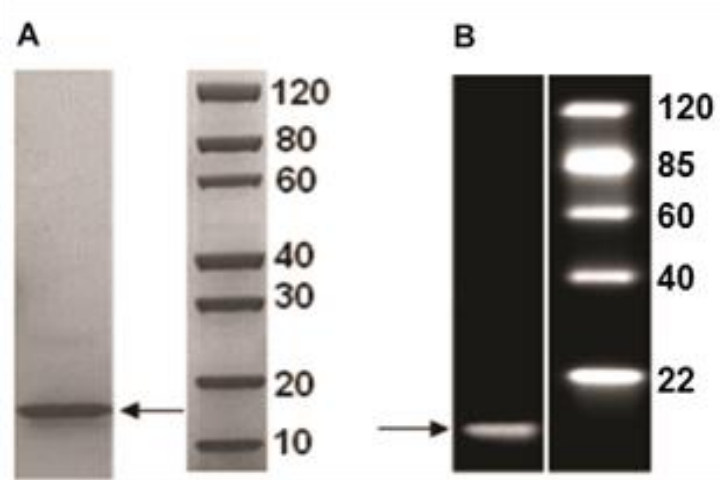

Supplement: Supplementary file 2 — Additional file 2: Figure S2. Expression of the PfCap380 peptide antigen. An image of an SDS-PAGE protein gel shows the migration of the purified His-tagged-PfCap380 peptide band in A (arrow). Western blot analysis shows the same peptide fragment recognized by an anti-His antibody in panel B (arrow). Protein molecular weight markers (10-120 or 22-120 kDa) are indicated. These results were generated by GenScript and shown with their permission. [file 12936_2018_2277_MOESM2_ESM.pdf]

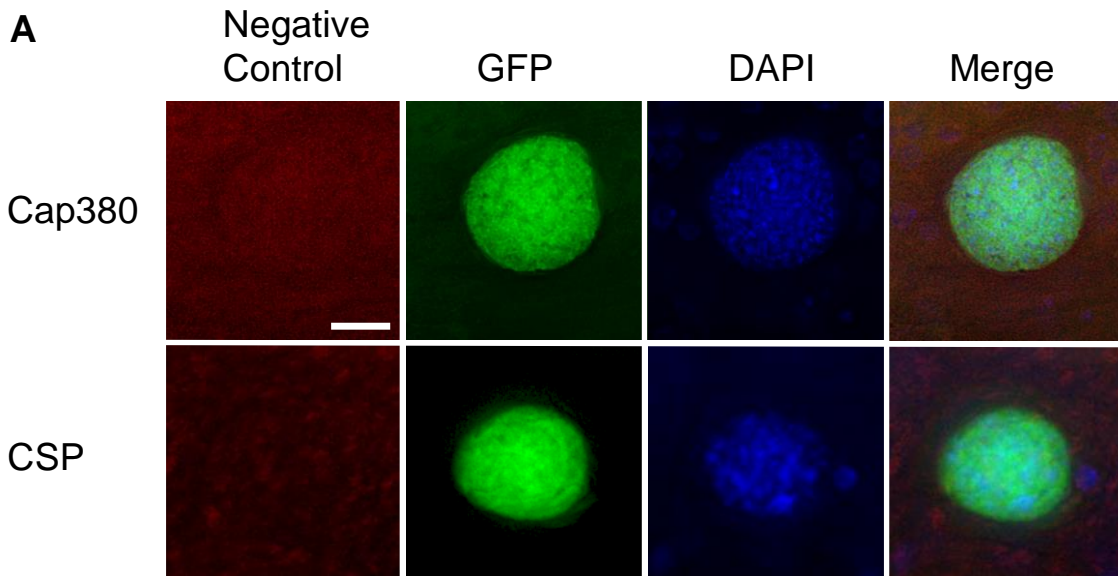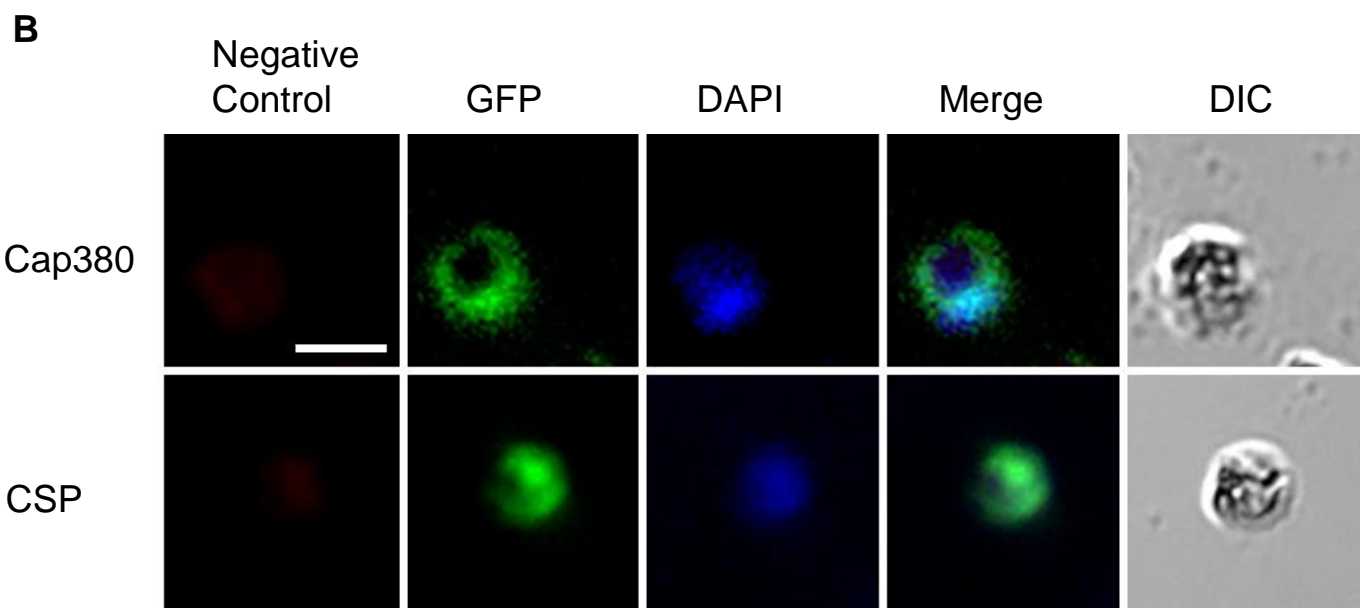

Supplement: Supplementary file 3 — Additional file 3: Figure S3. Negative control IFA on in vivo and in vitro oocysts. IFA were performed as described except no primary antisera was used to test secondary antibodies for non-specific binding. Secondary antibodies were used to label midgut oocysts (A) or in vitro oocysts (B) and show expression of GFP in green and DAPI nuclear staining in blue. The merged image of the three separate channels is shown. DIC images were taken for in vitro but not in vivo oocysts due to challenges in imaging midgut tissue. Scale bars = 10 μm. For midgut oocysts, negative control antibodies used were Alexa Fluor 594 anti-rabbit for Cap380 and Alexa Fluor 647 anti-mouse for CSP. For in vitro oocysts, negative control antibodies used were Alexa Fluor 594 anti-rabbit for Cap380 and Alexa Fluor 594 anti-mouse for CSP. [file 12936_2018_2277_MOESM3_ESM.pdf]

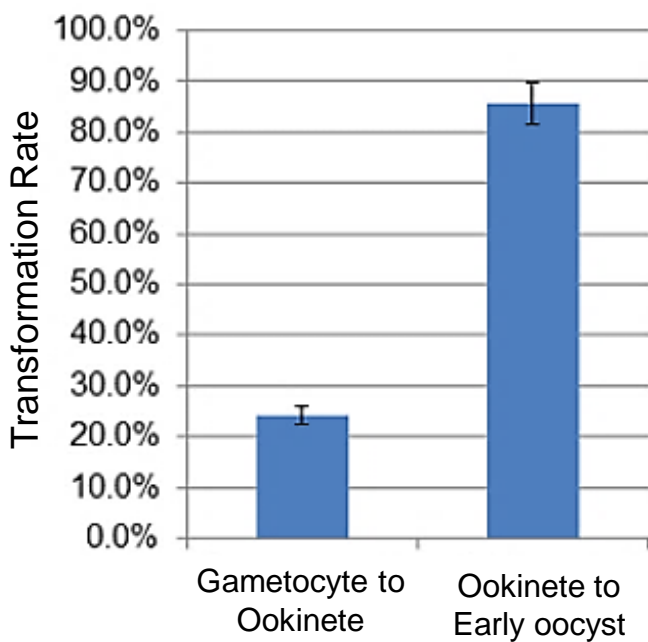

Supplement: Supplementary file 4 — Additional file 4: Figure S4. Transformation rates between parasite stages. The graph shows the transformation rates for gametocyte to ookinete stages and ookinete to early oocyst stages. The gametocyte to ookinete transformation rate was determined by counting gametocytes and ookinetes in a hemocytometer. The ookinete to oocyst transformation rate was determined by counting oocysts in an 8-well-chamber slide that formed after seeding a known quantity of ookinetes. The values depict averages across three experiments and error bars represent standard deviation. [file 12936_2018_2277_MOESM4_ESM.pdf]

Cap380

GFP

DAPI

Merge

1

2

3

4

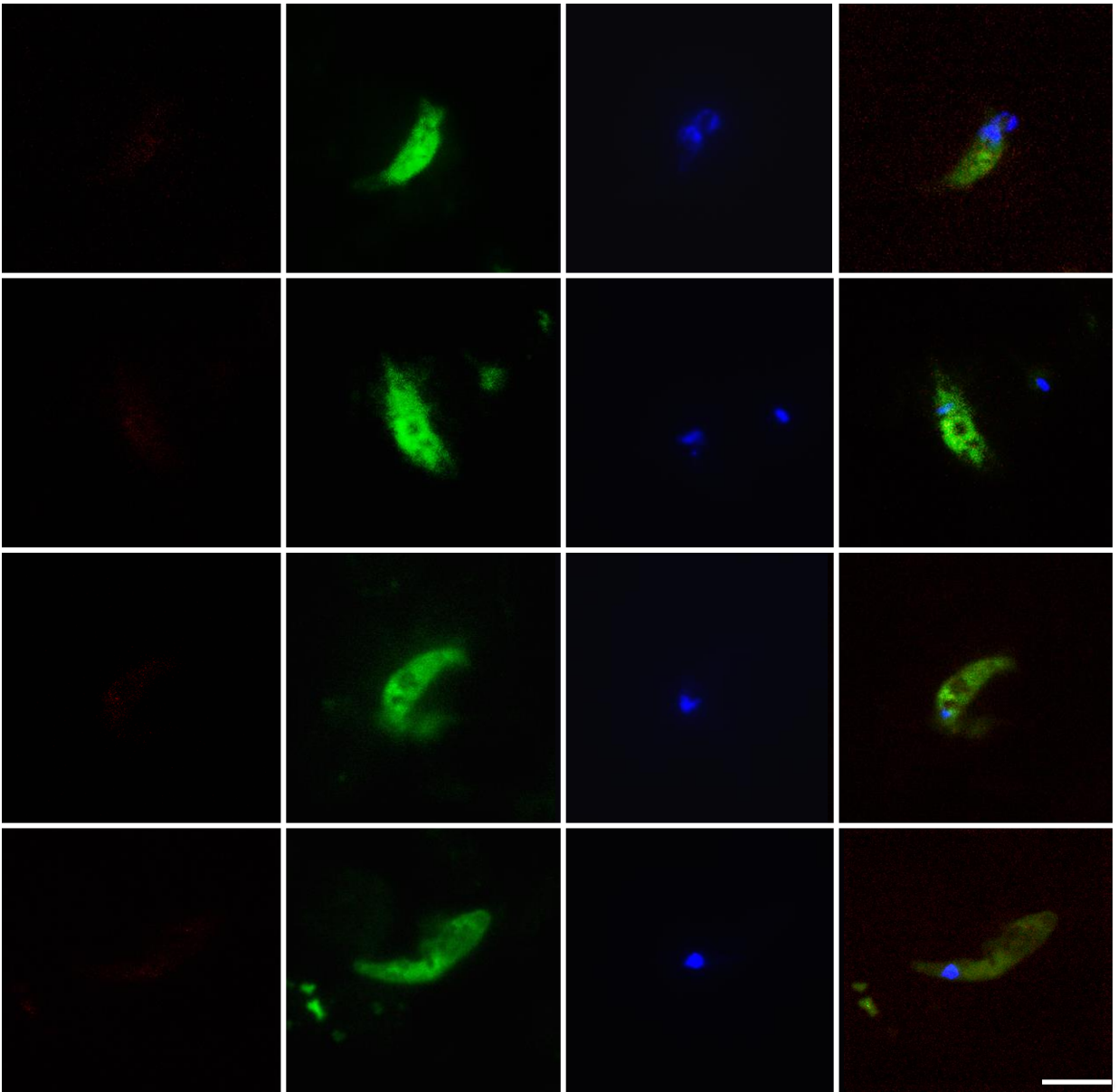

Supplement: Supplementary file 5 — Additional file 5: Figure S5. Ookinetes do not express PfCap380. IFA were performed using purified ookinetes with anti-PfCap380 antisera directly labeled with Alexa Fluor 594. Ookinetes (1-4) express GFP in green and nuclei stain with DAPI in blue but do not express PfCap380 (red). The merged image of the four separate channels is shown. Scale bar = 5 μm. [file 12936_2018_2277_MOESM5_ESM.pdf]

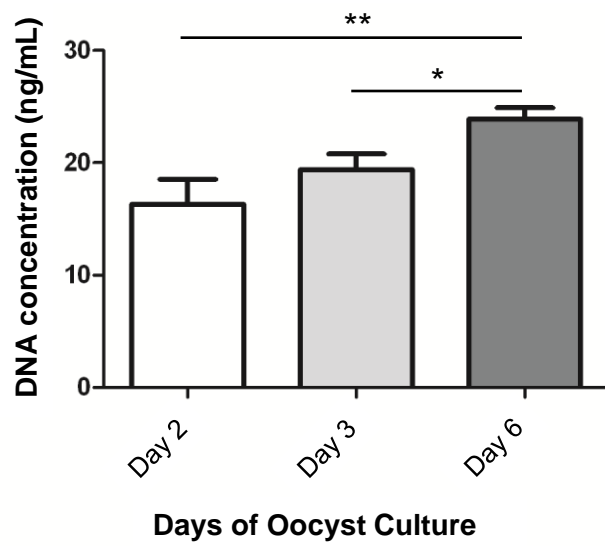

Supplement: Supplementary file 6 — Additional file 6: Figure S6. Oocyst DNA increases after six days of in vitro culture. After two, three, and six days in culture, oocysts were collected and DNA content was measured using a fluorescent nucleic acid stain specific for double-stranded DNA (dsDNA). dsDNA concentration was calculated by comparison to standards with known DNA concentrations. Mean values are shown for oocysts on days 2, 3 and 6. To determine the significance between groups, a One-way ANOVA and Tukey’s Test was performed. For significance, * = p < 0.05, ** = p < 0.01. The experiment was performed in triplicate, and error bars represent standard deviation. [file 12936_2018_2277_MOESM6_ESM.pdf]
