# Supplementary material for: PfCap380 as a marker for Plasmodium falciparum oocyst development in vivo and in vitro
Source: Malar J. 2018 Apr 2;17:135. doi: 10.1186/s12936-018-2277-6 (PMC5880026; doi:10.1186/s12936-018-2277-6)
Supplement: Supplementary file 1 — Additional file 1: Figure S1. Sequence alignment of Plasmodium Cap380 orthologues. Amino acid sequence alignment of the PfCap380 proteins for several Plasmodium species was performed to identify similarities within the peptide immunogen region. In A, Asterisks = fully conserved residues, colon = conservation with strongly similar properties, period = conservation with weakly similar properties. In B, the percentages of similar, identical, or gap amino acids are shown for the PfCap380 antigen compared to Plasmodium species. PfCap380 shares most amino acid similarity with Pg, followed by Pv, Pb, and then Py. [file 12936_2018_2277_MOESM1_ESM.pdf]

A

```
Pf      -MIYVCSDSIILHENPYMVVKPIFTLKSYLYNVGTHIIDDYMSYKYPRKLKHLEYINIPF
Pb      KYLIFVDGFITLHANPYVVVNPVFTEDSYYYNSNVSQTDDYVSFMYSRKLKLFEFLELPT
Py      KYLIFVDGFITLDTNPYVVVNPIFTEDSYYYNSNVSQTDDYVSFMYSRKLKLFEFLELPT
Pv      EYQIEVIGLVELHADPYVVIKPIFTHSSYLYNTVIYTTDDFVAYKYPRKLKYWEFLRIPF
Pg      MYSVNVNGNILLDKDPYAVIKPIFTSDSYLYNTSISIYDDYVTYKYPRKPKYSEFLDLPI
               . : *. :** *::*:** .** **      **:::: * ** *  *:: :*

Pf      SRSIFKSNTIDLSLVHK----RVEEDHKVCTVYGLEFVKSYELIRVIISDIFNNEFVVNL V
Pb      TQKLFKDNTMTITTEQL-----IHYQEETHNIYLLTLVKTLNLTKVIIRDIFNNEFEINI D
Py      TQMLFKDNTMTITTEQL-----IHYQEETHNVYLLTLVKTLNLTKVIIRDIFNNEFEVNI D
Pv      SKGLFKDKALTLRTVQCNTGRRLGDGAKAHCVYSLVLVKSCTLVRVVVRDLYGNEYEVSL D
Pg      SKNIFKGDSLNMSVGNI-----NETNNKIDNVYVLKMVKSFNLLSLIIRDMFNNEYVADL T
        :: :**..:: :   :           :   :* * :**:  *  ::: *::.**:  .:
```

B

Species compared to PfCAP380	Similar amino acids	Identical amino acids	Gaps
P. gallinaceum	81/109 (74%)	52/109 (48%)	1/109 (<1%)
P. vivax	73/109 (66%)	50/109 (46%)	4/109 (3%)
P. berghei	71/110 (64%)	51/110 (46%)	7/110 (6%)
P. yoelii	70/110 (63%)	53/110 (48%)	7/110 (6%)
